# Fiber optical imaging of astroglial calcium signaling in the respiratory network in the working heart brainstem preparation

**DOI:** 10.3389/fphys.2023.1237376

**Published:** 2023-08-24

**Authors:** Charlotte Tacke, Anne M. Bischoff, Ali Harb, Behnam Vafadari, Swen Hülsmann

**Affiliations:** Department of Anesthesiology, University Medical Center Göttingen, Göttingen, Germany

**Keywords:** glia, catecholaminergic signaling, neuronal control of breathing, norephinephrine, brainstem

## Abstract

The neuronal activity in the respiratory network strongly depends on a variety of different neuromodulators. Given the essential role of astrocytes in stabilizing respiratory network activity generated by neurons in the preBötzinger complex (preBötC), our aim was to investigate astrocytic calcium signaling in the working heart brainstem preparation using fiber-optical imaging. By using transgenic mice that express GCaMP6s specifically in astrocytes, we successfully recorded astrocytic calcium signals in response to norepinephrine from individual astrocytes.

## Introduction

Respiratory neurons in the ventrolateral medulla are closely intertwined with astrocytes that play an important role in stabilizing the autonomous activity of this network (Hulsmann et al., 2000; Grass et al., 2004; Schnell et al., 2011; Sheikhbahaei et al., 2018; Tacke et al., 2023). Astrocytes provide essential support to the neuronal network by controlling neurotransmitter homeostasis, ensuring proper neurotransmission (Gomeza et al., 2003; Szoke et al., 2006; Eulenburg and Hülsmann, 2022). Furthermore, various neuromodulators evoke intracellular calcium elevations in astrocytes, which may impact respiratory activity in turn (Funk et al., 2008; Härtel et al., 2009; Huxtable et al., 2010; Schnell et al., 2016). However, whether spontaneous calcium activity influences or drives respiratory neuronal activity is still a subject of ongoing discussion (Schnell et al., 2011; Okada et al., 2012; Oku et al., 2016; Tacke et al., 2023). Here, we built and tested a fiber optical setup to record and analyze astrocytic calcium signaling in the intact respiratory network of the working heart brainstem preparation. One aim of the study was to test whether calcium signals from individual astrocytes can be recorded with an imaging fiber.

## Materials and methods

### Breeding of mice

Animals were hosted and bred in the animal facilities of the University Hospital Göttingen. This study was carried out in accordance with the guidelines for the welfare of experimental animals issued by the European Communities Council Directive (2010/63/EU) and with the German Protection of Animals Act (Tierschutzgesetz; TierSchG). All procedures were approved by the Niedersächsische Landesamt für Verbraucherschutz und Lebensmittelsicherheit (LAVES).

### Induction of GCaMP6s expression in astrocytes

For calcium imaging, we used Aldh1l1-GCaMP6s mice [Tg (Aldhll1-cre/ERT2)02Kan; 129S-B6J.Cg-Gt (ROSA)26SorTM96(CAG−GCaMP6s)He/MwarJ], which allow for conditional and tamoxifen-inducible expression of GCaMP6s in astrocytes (Madisen et al., 2015; Winchenbach et al., 2016; Tacke et al., 2023). These mice were crossbred to receive double transgenic offsprings. Mice were between 9 and 20 months old, either heterozygous or homozygous for the Cre-recombinase and homozygous for the floxed-GCaMP6s-gene. For induction of GCaMP6s expression mice were intraperitoneally injected with tamoxifen (10 mg/mL dissolved in corn oil, 100 mg/kg body weight) once a day for two to five consecutive days at least 14 days prior to experiments. Additionally, mice that express EGFP under the control of the hGFAP-promoter (TgN (GFAP-EGFP)GFEC-FKi) were used for initial tests of the resolution and quality of the imaging system.

### Working heart brainstem preparation

Experiments were performed in the acute working heart brainstem preparation (WHBP, Paton, 1996; Paton et al., 2022). After an overdose of isoflurane anesthesia, confirmation of apnea and the absence of the nociceptive withdrawal reflex, they were bisected below the diaphragm for exsanguination. Furthermore, the cerebellum was removed and a decerebration at the parafollicular level was performed. The descending aorta was cannulated and retrogradely perfused at a flow rate of 16–20 mL/min using a peristaltic pump (Watson Marlow) with carbogen-saturated (95% O_2_, 5% CO_2_) artificial perfusion medium: The composition of the solution was (in mM): 125 NaCl; 25 NaHCO_3_; 2.5 CaCl_2_; 1.25 MgSO_4_; 1.25 KH_2_PO_4_; 5 KCl; 10 glucose, pH 7.4 at 32°C. For the optimization of the osmotic pressure the oncotic agent Ficoll (1.25%; Sigma–Aldrich) was supplemented. The perfusion pressure was monitored using the second lumen of the double-lumen perfusion catheter. The preparation was placed in a recording chamber and at the beginning of the experiment, the flow of the perfusate was adjusted to observe uniform phrenic nerve discharges. Thereafter, no further adjustment was allowed. A thoracic phrenic nerve (PN) was prepared and phrenic nerve activity (PNA) was recorded using a custom-made borosilicate glass suction electrode. PNA signals were amplified and band-pass filtered (0.25–2 kHz) using a custom-made amplifier (electronic workshop of the department of physiology, Göttingen). Signals were digitized by a PowerLab 8/30 (AdInstruments) and stored on a windows 10 PC.

### Design of the fiber optical imaging setup and calcium measurements

**FIGURE 1:**
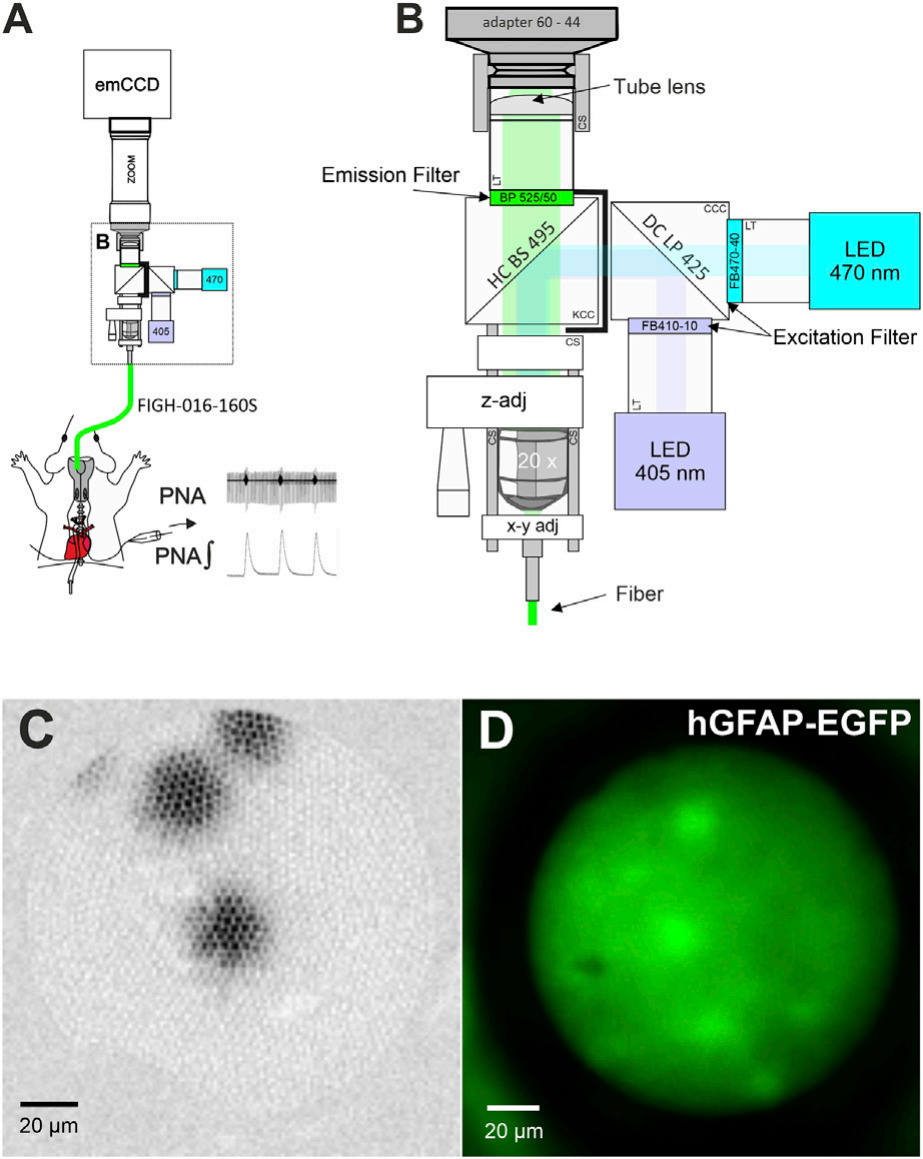
A fiber optical setup for imaging. **(A)** Schematic drawing of the fiber scope setup used with the working heart brainstem preparation. **(B)** Magnified view of core of the fiber scope. z-adj: The LINOS Microbench Z-Fine adjustment Tool is used to focus just above the surface of the imaging core of the fiber. y-x adj: The fiber can be centered on the CCD-chip using a fle.X-plate (Excelitas Technologies). See Table 1 for details of major parts. Adapter 60–44: video adapter (Zeiss); LT: lens tube system parts; CS: 30 mm cage system component; HC BS 495 and DC LP 425: Dichroic mirrors (beam splitter); CS: Lens Tube Flexure Sleeve Coupler; CCC: compact cage cube; KCC: kinematic cage cube (Thorlabs). Graphic is not to scale. **(C)** Image from three 10 µm fluorescent microspheres for demonstration of the image quality. **(D)** Image of astrocytes in a WHBP from a transgenic mouse expressing EGFP under the control of the human GFAP promotor [Tgn (hGFAP-EGFP)].

The fiber scope was assembled from parts of the LINOS^®^ Microbench system (Excelitas Technologies), the Thorlabs 30 mm Cage and SM1 Lens tube systems (Thorlabs; see Figure 1; Table 1). Two collimated LEDs, 470 nm (KSL-70; Rapp Optoelectronics) and 405 nm (M405L4–405 nm; LA1951-A; Thorlabs) were coupled to the scope via a Compact 30 mm Cage Cube (Thorlabs) using a long pass dichroic mirror (425 nm Cut-On; DMLP425R Thorlabs). Excitation light was filtered by bandpass filters BP470-40 (Zeiss) and FB410-10 (Thorlabs), respectively and coupled into the emission path via a HC BS 495 Beamsplitter (Semrock) mounted in a kinematic 30 mm Cage Cube (Thorlabs). The emitted light was band pass filtered (525/50 ET Bandpass; Chroma). The image of the fiber was projected to an emCCD camera with 128 × 128 pixels (Ixon 860, Andor) via a tube lens (Thorlabs, L1608-A; f = 75.0 mm) and a zoomable video adapter (0.5x to 2.4x; Zeiss; 452984-0000-000, Zoom 44, ENG 1/2”). The mechanical adapter for connecting the camera to the zoom was custom made by the workshop of the university medical center. The imaging fiber FIGH-016-160S (Fujikura) with 1,600 picture elements (Figure 1C) was focused with ×20 objective (EC Plan-Neofluar ×20/0,50, Zeiss) using a LINOS^®^ Microbench Z-Fine adjustment Tool M and centered to the optical path through a fiber optic adapter (N12.5 FC) and a fle.X-plate XY Steel (Excelitas Technologies).

**TABLE 1:** List of major parts that were used to assemble the fiber scope.

Parts	Specification	Manufacturer
Imaging fiber	FIGH-016-160S	Fujikura
Camera	IXON 860; 128 × 128 px; emCCD	Andor
Video Zoom	452984-0000-000; 0,5× ... 2,4×	Zeiss
Tube lens	L1608-A; f = 75.0 mm	Thorlabs
LED (470 nm)	KSL-70	Rapp Optoelectronics
LED (405 nm)	M405L4-405 nm; LEDD1B- T-Cube	Thorlabs
Dichroic Mirror (ex)	Long pass 425 nm Cut-On; DMLP425R	Thorlabs
Dichroic Mirror (em)	HC BS 495	Semrock
Filter (ex)	BP470-40	Zeiss
Filter (ex)	FB410-10	Thorlabs
Filter (em)	525/50 ET Bandpass	Chroma
Filter housing (ex)	Compact 30 mm Cage Cube	Thorlabs
Filter housing (em)	Kinematic 30 mm Cage Cube	Thorlabs
Objective	EC Plan-Neofluar 20x/0,50	Zeiss
Imaging software	Imaging workbench 6.1	Indec Biosystems
Fiber focusing	LINOS^®^ Microbench Z-Fine adjustment Tool M	Excelitas Technologies
Fiber centering	fle.X-plate XY Steel	Excelitas Technologies

GCaMP6s was excited with two wavelengths: 470 nm was used for the Ca^2+^-dependent signal and 405 nm for the Ca^2+^-independent isosbestic control measurements (Kim et al., 2016). For wavelength control and image acquisition, Imaging Workbench 6.1 (Indec Biosystems) was used and ratio image pairs were captured at approximately 8 Hz. The Software controlled the LED drivers (KSL-70 for the 470 nm LED and LEDD1B-T-Cube for the 405 nm LED, respectively) via a TTL signal provided by the parallel out of a Windows 10 PC. Image sampling time was set to 40 ms.

For positioning the bare end of the fiber in the ventral respiratory column (VRC), we used a modified “*InVivo* Neuro Pixels Junior unit” micromanipulator (Luigs&Neumann). Since the cerebellum was removed, the fourth ventricle and the obex were used as landmarks for navigating the fiber to the VRC based on the information from the mouse brain atlas (Paxinos et al., 1985). First the fiber was placed on the surface of the dorsal medulla (1-1.5 µm rostral to the obex, 1.2-1.5 µm lateral to the midline) and then carefully advanced vertically into tissue to find fluorescence (1-1.5 µm).

### Fiber size determination

For infinity corrected ×20 Zeiss objectives, the magnification of the objective is 20 if the focal length (f) of the tube lens is 164.5 mm. However, we used a tube lens with a focal length of 75 mm, therefore the magnification of the objective was ×9.1. The total magnification of the system further depends on the magnification of zoomable video adapter, which can be changed from 0.5x to 2.4x. Thus, the range of the magnification of the system can be changed between ×4.6 and ×21.9. The IXON camera has 128 × 128 pixel with a pixel size of 24 × 24 µm. Therefore, the field of view of the camera can be changed stepless between 140.4 × 140.4 µm and 673.8 × 673.8 µm. Based on this information, image circle diameter of the fiber can be measured. The fiber in our experiment had an image circle diameter of 143 µm.

We used Fluoresbrite^®^ Yellow Green Microspheres (10 μm; Polysciences) to confirm that the illumination spot was sufficiently large to fill the entire fiber (Figure 1C). Additionally, we tested the system with EGFP-labeled astrocytes to confirm single cell resolution. (Figure 1D).

### Drugs

Electrolytes for perfusion solution (see above) were purchased from Sigma-Aldrich (Taufkirchen, Germany) or Merck chemicals (Darmstadt, Germany), Tamoxifen, Ficoll^®^ 70 and norepinephrine from Sigma-Aldrich.

### Data analysis

Image series were stored on a PC in the original format (.axi) of the imaging workbench software (Indec Biosystems). The ratio images were exported to tiff-format and 3D median filtered (3x 3x 5x) using the Fiji distribution of ImageJ (Schindelin et al., 2012). The ROI-manager tool was used to analyze the fluorescent signal from the entire image core of the fiber (regions of interest (ROI) diameter = 143 µm) or from individual cells (ROI diameter <20 µm). Ratio image data is given as arbitrary units (a.u.). For graphical presentation, the timing of image acquisition was exported to txt-format from the statistics window of imaging workbench and imported to IGOR Pro software (WaveMetrics). Figures were assembled using CorelDraw (Corel Corporation).

Data are expressed as mean ± SD. Statistical comparison was performed with the SigmaPlot software (Systat). Statistical significance (Mann-Whitney Rank Sum Test) was assumed if *p* < 0.05.

## Results

**FIGURE 2:**
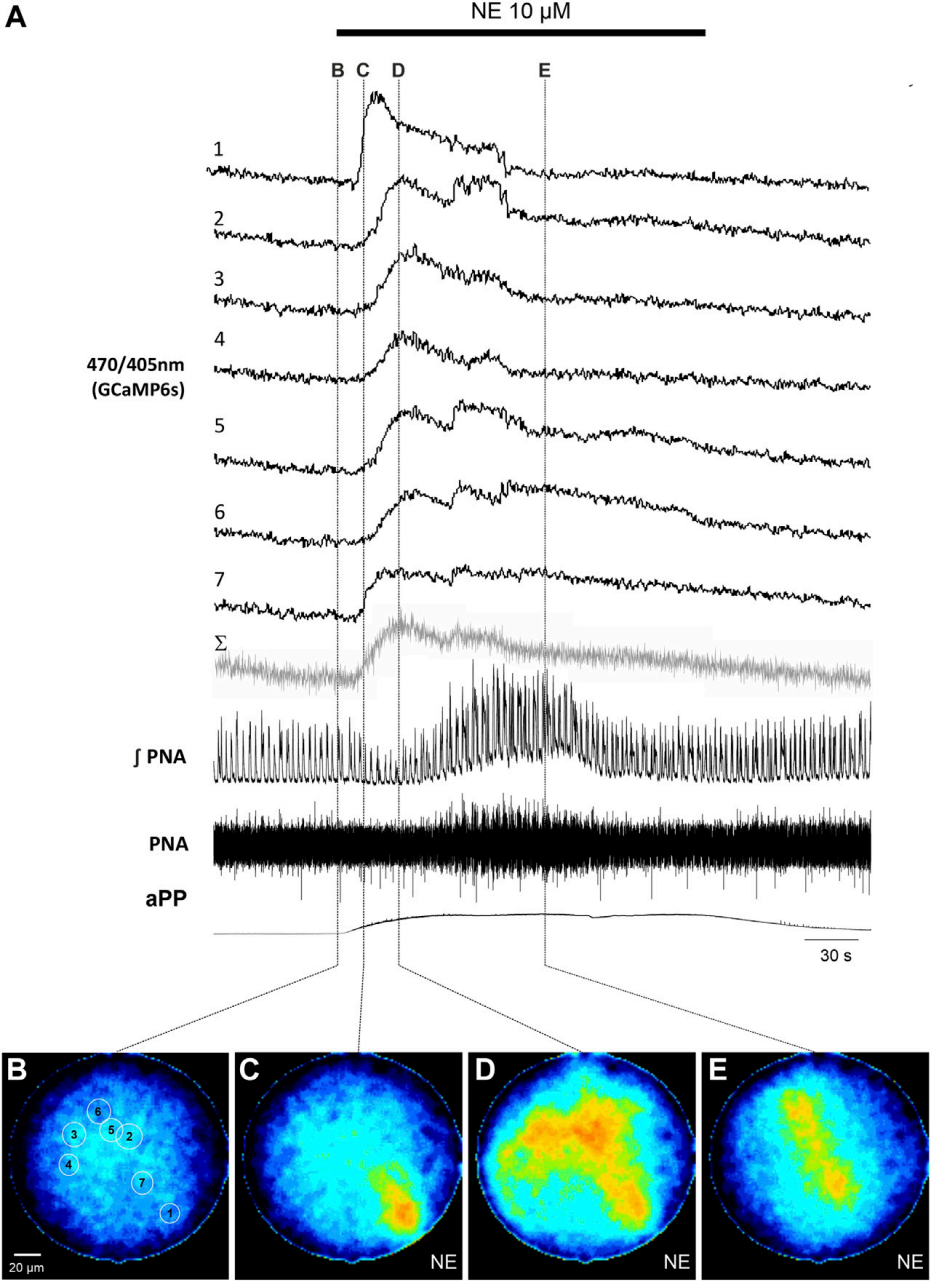
Norepinephrine (NE)-induced calcium signals in astrocytes in the WHBP. **(A)** Original traces from the 7 cells [circular ROI, see **(B)**] together with phrenic signal (integral and raw) and the apparent perfusion pressure (aPP). The gray trace (Σ) represents the summated signal across the entire fiber. The bar indicates the time NE was applied to the perfusate. **(B–E)** False color coded 470/405 nm ratio images at different time points before **(B)** and after **(C–E)** the application of NE (10 µM). Note: The withdrawal of NE from the circulation can be nicely monitored by the drop of the perfusion pressure.

**FIGURE 3:**
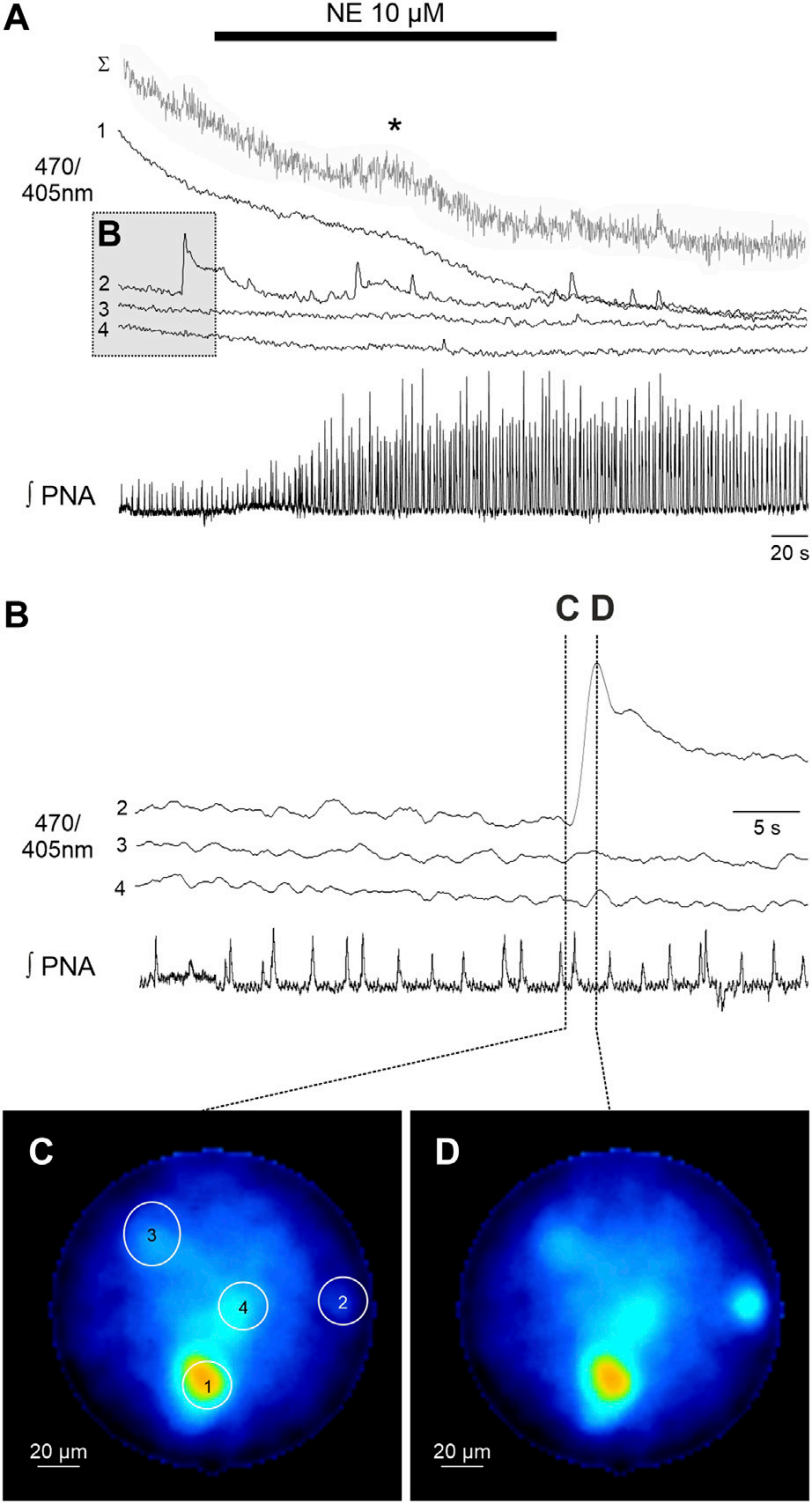
Spontaneous calcium signals in astrocytes recorded with the fiber microscope in the WHBP. **(A)** Original GCaMP6s measurements of 4 individual astrocytes (470/405 nm) together with phrenic signal (integral). The gray trace (Σ) represents the summated signal across the entire fiber. Cell 2 shows spontaneous calcium signals, while in summated signal a delayed response (*) to norepinephrine (NE 10 µM) is visible. **(B)** Detailed representation of the calcium signal of cells two to four prior to the administration of NE. Note that there are no calcium signals in phase with the PNA inspiratory burst. **(C,D)** False color coded 470/405 nm ratio images at different time points before **(C)** and during **(D)** the spontaneous calcium signal of cell 2.

To induce reliable calcium signals in astrocytes, 10 µM norepinephrine (NE) was added to the perfusion. NE induced an increase of the apparent perfusion pressure (aPP) of 49.4 ± 26.3 mmHg (*n* = 11). Additionally, there was also a variable increase of the phrenic nerve activity (PNA) with larger amplitudes and an increase of the respiratory rate (Figures 2, 3). The peak of the PNA-response followed the initial increase of the aPP after 104.7 ± 5.2 s (*n* = 11). In four recordings, the increase of the aPP was accompanied by a short period of reduced PN activity (Figure 2).

Analysis of the emission of the full image core of the fiber revealed an increase of the calcium activity of the 470/405 nm ratio signal in 10 out of 11 recordings/preparations. Interestingly, the astrocytic calcium rise preceded the increase of PNA in 8 preparations (average delay 57.1 ± 21.0 s; *p* = 0.008; Mann-Whitney Rank Sum Test).

In a second step we analyzed the calcium responses in more detail. In 4 of the preparations individual cells could be identified by their individual, non-uniform calcium signaling (Figures 2, 3). Since the individual signals show different timing and duration, we assume that these signals represent the calcium response of individual cells. It is important to note that these individual signals are clearly distinguishable from the integrated emission of the full fiber (gray trace in Figures 2A, 3A). Furthermore, in 2 preparations the analysis of the different cells revealed spontaneous calcium signals in individual cells (Figure 3). Calcium signals that were phase locked to the inspiratory phrenic bursts were not observed (Figure 3).

## Discussion

In this paper, we were able to prove that calcium imaging of astrocytes in the working heart brainstem preparation is possible by using a commercially available imaging fiber and a custom-made fiber-coupled microscope. It was possible to discern NE-induced and spontaneous calcium signals from individual cells. Furthermore, we also showed that NE-induced calcium signals in astrocytes precede the NE-induced increase of the respiratory network activity, which has been described in rodents (Viemari, 2008).

Recording calcium signals from individual cells represents a clear advantage compared to the classical fiber photometry, which can only capture ‘bulk’ or population-level calcium (Ca^2+^) activity (Gunaydin et al., 2014). This is particularly important when analyzing spontaneous astrocytic signals, which might not be detected in the bulk signal (see Figure 3).

Interestingly, in four of the experiments there was a short period of a reduced PNA, which started simultaneously with the NE-induced increase of the perfusion pressure (aPP). Activation of arterial baroreceptors by the elevated aPP or directly by NE (Holmes and Ledsome, 1984), would negatively modulate the network in the VRC (Simms et al., 2007; Barnett et al., 2021). After adaptation of the baroreflex, the neuron-mediated stimulation of the network by NE will become dominant (Zanella et al., 2014; Tacke et al., 2023). Further experiments are required to test if the depression is eventually a response of the network to the astrocytic calcium rise and release of an inhibitory gliotransmitter (Gourine et al., 2010; Forsberg et al., 2017).

In response to NE, individual cells showed different delay and signal duration. These signals result most likely from the soma of the astrocyte, since it is known that NE induces strong somatic calcium-signals (Schnell et al., 2016). However, with the fiber imaging system we were not able to discriminate between different parts of the cells, thus making it impossible to distinguish calcium signals of processes from somata of individual cells. Thus, during the peak of the NE-response (Figure 2D), we observe a rather blurred image consisting of the overlapping calcium signals in somata and processes of neighboring astrocytes. Indeed, subcellular structures were not visible in the hGFAP-EGFP mice, in which high baseline fluorescence usually allows this discrimination using epifluorescence or 2P-imaging (Grass et al., 2004; Hirrlinger et al., 2004). Norepinephrine was applied to the perfusate and therefore reached the medulla via the blood stream. We cannot exclude that the perfusion with the artificial solution is altering the blood brain barrier (BBB) integrity, but even if this was intact, it is known that brain capillary endothelial cells and astrocytes express norepinephrine transporter that allow NE to enter the brain (Wakayama et al., 2002; Inazu et al., 2003; Schnell et al., 2016). The apparent delay between the vascular response and the astrocytic calcium signal is in favor of an intact BBB.

We used a ratiometric approach for the calcium imaging with two wavelengths for excitation. 470 nm allows the detection of calcium-induced fluorescence change and 405 nm, which is very close to the isosbestic point of GCaMP6s (Dana et al., 2019). Under baseline condition, individual cells, however, were not always distinguishable (Figure 2B) but appeared only after application of NE (Figure 2). One reason is probably that the lateral resolution is limited by the size of the fiber cores (2 µm). Since at its isosbestic point the emission of GCaMP6s (Ca^2+^-free or Ca^2+^-saturated) is below 10% of the maximal fluorescence of the Ca^2+^-saturated GCaMP6 at 470 nm (Dana et al., 2019), low baseline calcium levels might not be detectable. Thus, it can be concluded that the fiber imaging setup requires a higher signal to noise ratio for the detection of (baseline) calcium levels as e.g., two-photon imaging, where most astrocytes are visible at rest (Tacke et al., 2023). One possible source of noise is autofluorescence of the imaging fiber (Udovich et al., 2008). The transmissibility (t) of the fiber and the objective (t @410 nm 83.8% vs. t @470 nm 89.9%), however, should not be a limitation. Nevertheless, we could prove that NE-induced as well as spontaneous calcium signals can be recorded with the setup.

The imaging fiber has a relatively short working distance <100 µm (Feng et al., 2022), thus, the fiber has to be inserted into the tissue and could potentially damage critical structures. We cannot exclude that this manipulation induces calcium signals in astrocytes (see high baseline signal in cell 1, Figure 3A) in response to the mechanical stimulus (Lee et al., 2015). For this project, we limited the size of the imaging fiber to an outer diameter of about 200 µm to minimize the impact of the fiber when inserting into the tissue to reach the ventral respiratory column. Further trials with larger fibers and a higher number of cores need to be tested.

## Conclusion

Utilizing an imaging fiber enables the measurement of calcium signals from individual astrocytes. This is particularly valuable when analyzing spontaneous calcium signals, which can be very heterogeneous in astrocytes (Schnell et al., 2011). Although the spatial resolution of the imaging fiber system is not as high as that for our two-photon microscope in acute brains slices (Schnell et al., 2016; Tacke et al., 2023), it allows deep tissue recordings in a complex preparation, such as the WHBP, where the complex intricate circuitry of the respiratory network remains intact.

## Data Availability

The raw data supporting the conclusion of this article will be made available by the authors, without undue reservation.
